# Comparative Analysis of Morphological and Functional Effects of ^225^Ac- and ^177^Lu-PSMA Radioligand Therapies (RLTs) on Salivary Glands

**DOI:** 10.3390/ijms242316845

**Published:** 2023-11-28

**Authors:** Benedikt Feuerecker, Andrei Gafita, Thomas Langbein, Robert Tauber, Christof Seidl, Frank Bruchertseifer, Jürgen E. Gschwendt, Wolfgang A. Weber, Calogero D’Alessandria, Alfred Morgenstern, Matthias Eiber

**Affiliations:** 1 Department of Nuclear Medicine, School of Medicine, Technical University of Munich, 81675 München, Germany; 2 Deutsches Konsortium für Translationale Krebsforschung (DKTK), Partnersite München, 69124 Heidelberg, Germany; 3 Department of Radiology, University Hospital, LMU Munich, 81377 München, Germany; 4 Department of Radiology, School of Medicine, Technical University of Munich, 81675 München, Germany; 5 Division of Nuclear Medicine and Molecular Imaging, The Russell H. Morgan Department of Radiology and Radiological Science, Johns Hopkins University School of Medicine, Baltimore, MD 21287, USA; 6 Department of Urology, School of Medicine, Klinikum Rechts der Isar, Technical University of Munich, 81675 München, Germany; 7 European Commission, Joint Research Centre (JRC), 76344 Karlsruhe, Germany

**Keywords:** xerostomia, PSMA, Actinium-225-PSMA-617, mCRPC, radioligand therapy, salivary glands, tumor sink effect

## Abstract

Most Prostate Specific Membrane Antigens (PSMAs) targeting small molecules accumulate in the salivary glands (SGs), raising concerns about SG toxicity, especially after repeated therapies or therapy with ^225^Ac-labeled ligands. SG toxicity is assessed clinically by the severity of patient-reported xerostomia, but this parameter can be challenging to objectively quantify. Therefore, we explored the feasibility of using SG volume as a biomarker for toxicity. In 21 patients with late-stage metastatic resistant prostate cancer (mCRPC), the PSMA volume and ligand uptake of SG were analyzed retrospectively before and after two cycles of ^177^Lu-PSMA (LuPSMA; cohort A) and before and after one cycle of ^225^Ac-PSMA-617 (AcPSMA, cohort B). Mean Volume-SG in cohort A was 59 ± 13 vs. 54 ± 16 mL (−10%, p = 0.4), and in cohort B, it was 50 ± 13 vs. 40 ± 11 mL (−20%, p = 0.007), respectively. A statistically significant decrease in the activity concentration in the SG was only observed in group B (SUV_mean_: 9.2 ± 2.8 vs. 5.3 ± 1.8, p < 0.0001; vs. A: SUV_mean_: 11.2 ± 3.3 vs. 11.1 ± 3.5, p = 0.8). SG volume and PSMA-ligand uptake are promising markers to monitor the SG toxicity after a PSMA RLT.

## 1. Introduction

The treatment of metastatic castration-resistant prostate cancer (mCRPC) remains a major challenge. A prolonged overall survival with the radiopharmaceutical ^177^Lu-PSMA-617 has been recently proven in a phase III clinical trial compared to the standard of care (median OS 15.3 vs. 11.3 months) [1]. However, primary or secondary radioresistance to ^177^Lu-PSMA (LuPSMA) limits its effect [2]. It has been proposed that targeted alpha therapy (TAT) has the potential to overcome the radioresistance of beta emitters through its higher linear energy transfer [3,4]. TAT has been proven to be more effective than beta emitters in preclinical studies as it induces DNA double-strand breaks [3]. 

The alpha emitter Actinium-225 (^225^Ac) has been recently used for the PSMA-targeted treatment of mCRPC, and promising results have been reported using ^225^Ac-PSMA-617 (AcPSMA) [3,4,5]. However, xerostomia is a major limiting side effect for AcPSMA, which can lead to the discontinuation of treatment [4,6]. Deterioration of salivary function is a clinical problem described after an external beam radiation treatment [7,8] and after a radioiodine treatment [9,10,11]. Its extent has been related to the absorbed dose based on the data of external beam radiation therapy [12]. For alpha emitters, quantitative radiation dosimetry is not trivial, given the lack of direct gamma emissions. Therefore, a quantitative measurement of delivered dose to the salivary glands (SGs) is highly challenging. Dose estimations can be made based on the dosimetry of LuPSMA treatment and serial PET measurements. Salivary gland scintigraphy provides an objective measure to quantify SG function and has been reported as a tool to assess SG function in patients with thyroid diseases [13,14,15,16] and mCRPC [17]. Furthermore, an indirect measurement of the effects of radiation on SG can be made, based on pre- and post-therapeutic staging scans such as PSMA PET combined with morphological imaging. 

Therefore, our aim of this retrospective analysis was to investigate the potential correlates in the morphological and molecular PET imaging of clinically observed xerostomia. Pre- and post-treatments hybrid PET imaging in patients who have undergone ^225^Ac-PSMA-617 radioligand treatment (RLT) and ^177^Lu-PSMA-I&T RLT were compared. We hypothesize that decreases in SG volumes and PSMA-ligand uptake (a) are dependent on the type of radiation (alpha vs. beta) and (b) are related to xerostomia.

## 2. Results

### 2.1. Volumetric Changes in Salivary Glands before and after LuPSMA and AcPSMA RLTs

**Figure 1:**
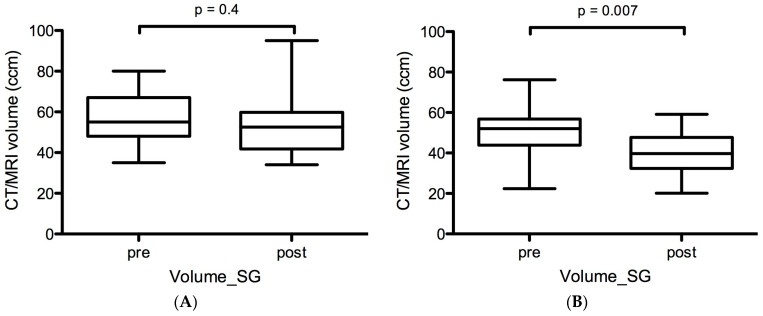
Morphological changes in SG volume based on CT/MRI quantification after ^177^Lu-PSMA (**A**) and ^225^Ac-PSMA-617 (**B**).

In cohort A (before vs. after LuPSMA RLT), no significant volumetric size changes were observed: the mean Volume-SG of the SG was 59 ± 13 vs. 54 ± 16 mL (p = 0.4, Figure 1A). Mean relative and absolute changes in Volume-SG were 10% and 5 mL. 

In contrast, a highly significant decrease in volumes was observed in cohort B (before vs. after AcPSMA RLT): the mean Volume-SG was 50 ± 13 mL vs. 40 ± 11 mL (*p* = 0.007, Figure 1B). Mean relative and absolute changes in Volume-SG were 20% and 10 mL.

### 2.2. Functional Changes in PSMA-Ligand Uptake before and after LuPSMA and AcPSMA RLTs

**Figure 2:**
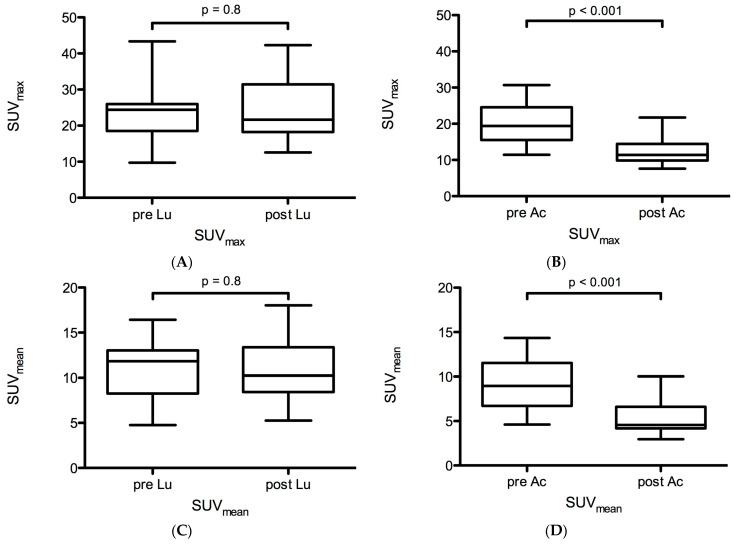
Changes in SUV_max_ (the total of submandibular and parotid glands) after ^177^Lu-PSMA RLT (**A**) and after ^225^Ac-PSMA-617 RLT (**B**), respectively, and change in SUV_mean_ (the total of submandibular and parotid glands) after ^177^Lu-PSMA RLT (**C**) and ^225^Ac-PSMA-617 RLT (**D**), respectively.

**Figure 3:**
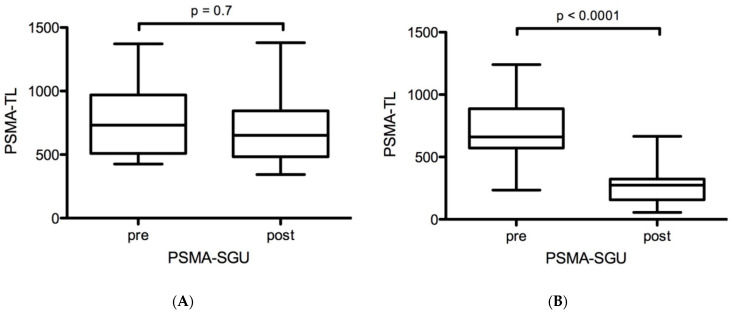
Changes in PSMA-SGU after ^177^Lu-177-PSMA RLT (**A**) and after ^225^Ac-PSMA-617 RLT (**B**), respectively.

In cohort A, no significant changes in PSMA-ligand uptake were observed: the mean SUV_max_ and SUV_mean_ were 23.8 ± 7.7 vs. 24.7 ± 8.7 (*p* = 0.8) and 11.0 ± 3.3 vs. 10.8 ± 3.4 (*p* = 0.8), respectively (Figure 2A,C). Mean relative changes in SUV_max_ and SUV_mean_ were +3.8% and −1.8%. The mean PSMA-SGU was 757 ± 264 vs. 721 ± 316 (*p* = 0.7, Figure 3A). Mean relative and absolute changes for PSMA-SGU were −5% and −30 (Figure 3A).

In contrast, a highly significant decrease in PSMA-ligand uptake was observed in cohort B: the mean SUV_max_ and SUV_mean_ were 20.1 ± 5.4 vs. 12.3 ± 3.6 (*p* < 0.0001) and 9.2 ± 2.8 vs. 5.3 ± 1.8 (*p* < 0.0001), respectively (Figure 2B,D). Mean relative changes in SUV_max_ and SUV_mean_ were −38.8% and −42.4%. The mean PSMA-SGU was 711 ± 268 vs. 276 ± 162 (*p* < 0.0001). Mean relative and absolute changes for PSMA-SGU were −61% and −435 (Figure 3B).

### 2.3. Salivary Glands and Tumor Burden

**Figure 4:**
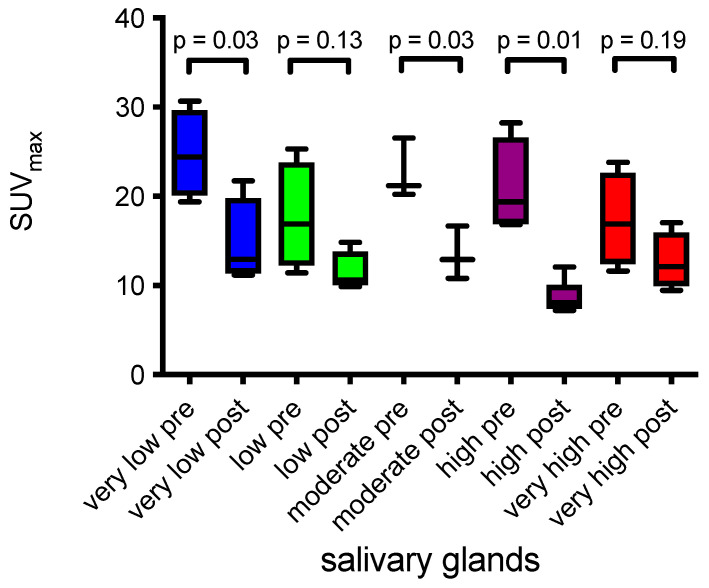
SUV_max_ of the SG stratified by tumor burden before (pre) and after (post) ^225^Ac-PSMA-617 RLT and also stratified by tumor load (colors indicate groups). Group moderate, *n* = 3, all other groups, *n* = 4.

Based on the five quartiles of pre-therapeutic whole body tumor burden, changes in the salivary gland SUV_mean_ and SUV_max_ pre- and post-AcPSMA were quantified. Statistically significant decreases in SUV_max_ of the SG were measured in groups with very low, moderate, high, and very high pre-therapeutic tumor burden (Table 1 and Figure 4). No correlation between SUV_mean_ of the SG and tumor burden was observed in the low and very high groups (Table 1). In each of these five tumor burden groups, no statistically significant changes in whole body tumor burden were observed post-AcPSMA. No significant changes in SUV_max_ and SUV_mean_ were observed in groups with very low, low, high, and very high tumor burden patients treated with LuPSMA.

## 3. Discussion

In this retrospective analysis, a treatment with one cycle of AcPSMA resulted in a significant decrease in morphological and functional surrogate parameters of salivary glands, which were assessed with PSMA PET. In contrast, no substantial differences could be observed after treatment with two cycles of LuPSMA in the same patients. 

The deterioration of the salivary gland function is a clinically relevant side effect of AcPSMA reported in the literature [3,6,18]. Our retrospective study is the first to present quantitative data from imaging to potentially link it with objective measures. For the external beam radiation treatment [7,19] of the neck, different reports on potential xerostomia using imaging as a quantitative measure are available. In an MRI study including 52 patients with squamous cell carcinoma of the neck, the volume of the parotid glands decreased by an average of 26% at 30 Gy and approx. 40% at 70 Gy [20]. In another study with 15 head and neck cancer patients, the median parotid volume loss was 28.1% (range: 5.9–53.6%) [21]. Furthermore, in a study with 18 patients irradiated with a radiation dose of 38.1 to 64.4 Gy, a reduction of the parotid glands by approximately 35%, was observed [22].

The evaluation of delivered doses of Actinium-225 to the salivary gland remains challenging because radiation doses depend on the microscopic distribution of the radioactivity within the tissue, which is currently unknown. Based on a dose assumption, an administration of 10 kBq/kg of ^225^Ac-PSMA-617 would result in a mean salivary gland dose of approximately 67 Gy [23]. For LuPSMA, data on the dosimetry of the salivary glands for both LuPSMA-617 [24,25,26] and LuPSMA-I&T [27] exist, resulting in a dose of 8.1–21.9 Gy to the salivary glands (after two i.v. injections of 7.4 GBq LuPSMA). 

In our retrospective analysis, Volume-SG was reduced by 10% in cohort A but by 20% in cohort B. Similarly, PSMA-SGU was reduced by −5% in cohort A but by −61% in cohort B. These data indicate that LuPSMA has only minor effects on the salivary glands, but AcPSMA induces profound physical and biological effects on the salivary glands. This is in line with the clinical observation that patients treated with LuPSMA rarely report a permanent xerostomia or request for a stop of treatment [28]. 

Based on the data presented here, both function (PSMA-SGU) and morphological size (Volume-SG) of the salivary glands decreased significantly after AcPSMA RLT. Considering the production of ca. 1 Liter/day of saliva (70% arising from the parotid, submandibular, and sublingual glands [29]), a reduction of ca. −20% (Volume-SG) to −61% (PSMA-SG) could hypothetically result in a daily production of ca. 390–800 mL of saliva. A range of 0.12–0.16 mL/min for salivary flow rate has been described as a critical range for patients and defines a clinically relevant hypofunction [30]. This would translate into a critical range of daily salivary production of approximately 172–230 mL. PSMA-SGU reduction after AcPSMA RLT reaches close to this critical range as shown by the above calculation. In fact, the relative morphological changes after AcPSMA RLT of the salivary glands were almost three times lower compared to the functional changes (Volume-SG −20% vs. PSMA-SGU −61%), and therefore, a reduction in Volume-SG may not fully explain the loss of salivary function. In summary, PSMA-SGU seems to correlate more closely to clinically observed xerostomia than Volume-SG and might be a more predictive parameter of salivary gland (dys)function. 

With respect to the tumor sink effect, controversial results have been reported after LuPSMA RLT. In mCRPC patients that were visually classified based on ^68^Ga-PSMA uptake, a decline in the salivary glands of 36–43% was observed [31]. Gafita et al. report a decrease in SUV_max_ in patients with a very high PSMA-VOL by an average of −26.6% [32]. Werner et al. report no correlation between salivary gland uptake and tumor volume in a study with 50 patients using ^18^F-DCFPyL PET [33]. Given the already relatively high tumor burden in our cohort, the observation of no additional tumor sink effect in the very high PSMA-TUB group compared to the low volume group might be explainable. In the study by Gafita et al., the patient group with a very high tumor burden had a Volume-SG of ≥1355 mL, which corresponds to the second quintile (1095–1610 mL) of our study (the very high tumor burden group of our study exhibited a Volume-SG of ≥4039 mL). However, a tendency towards a tumor sink was observed (Figure 4).

Xerostomia as a result of PSMA treatment is a known side effect, which is caused by a physiological tracer uptake [34,35,36]. It has been reported that xerostomia is less pronounced after the first cycles of ^177^Lu-PSMA RLT and in patients with a higher tumor burden due to the tumor sink effect [31,37,38]. Xerostomia was also described after a ^131^I-labeled MIP-1095 PSMA therapy as the second most common side effect after hematological toxicity [39] with ca. 25% of the patients demonstrating a dry mouth [40]. However, xerostomia was also reported in patients treated with other PSMA ligands at a high variability of frequency [2,41,42,43,44]. Initial studies with ^177^Lu-PSMA-617 reported that 2/56 patients showed xerostomia [45], while the frequency of grade 1 xerostomia reached up to 80% as per a report of a prospective phase 2 trial [34]. In a recent study including 30 patients using ^177^Lu-PSMA-617, CTCAE grade 2 xerostomia occurred in 17% of the patients [46]. On the other hand, the frequency of transient dry mouth symptoms in 26 patients treated with repetitive cycles of ^177^Lu-PSMA was 46% [28]. In patients treated with ^225^Ac-PSMA-RLT, data indicate a higher frequency and a pronounced impact on quality of life of xerostomia, leading to the request of treatment in up to 25% of patients [6]. Interestingly, our morphological data show that, at the initiation of the AcPSMA treatment, the salivary glands were already reduced compared to the beginning of the LuPSMA treatment (ca. 59 vs. 50 mL, −15%), pointing to the fact that LuPSMA treatment results in a slow decrease in salivary gland sizes. 

## 4. Materials and Methods

### 4.1. Patient Population

Data of mCRPC patients who underwent PSMA PET/CT or PET/MRI before and after ^177^Lu-PSMA-I&T—(LuPSMA) and ^225^Ac-PSMA-617—(AcPSMA) RLTs were retrospectively analyzed. Only patients who had comparable imaging data (which used similar PSMA-ligand pre- and post-treatments) with a sufficient coverage of the parotid gland were included.

First, 21 patients (cohort B), who were treated with AcPSMA as a salvage therapy after previous treatments (e.g., chemotherapy and the use of novel androgen receptor-targeted therapy) and who showed disease progression after LuPSMA RLT, were included. Tumor response and adverse events of these patients have been recently reported [6]. Second, out of these 21 patients, 15 patients (cohort A) were identified who underwent LuPSMA at our institution and for whom appropriate pairs of PSMA PET/CT or PET/MRI data (2 patients) were available. 

Patients’ xerostomia was graded on a three-point Likert scale (no to only mild xerostomia: grade 1; moderate symptoms with minor effects on daily life: grade 2; and severe xerostomia with major impacts on daily life/food or drink intake: grade 3). In total, nine patients had grade 1 xerostomia, six patients had grade 2, and six patients had grade 3 xerostomia.

Patient and treatment details for AcPSMA and LuPSMA are given in Table 2. All patients signed an informed consent and were treated under compassionate use after a discussion of an interdisciplinary tumor board. The present retrospective analysis was approved by the local ethics committee under the reference number of 115/18S.

### 4.2. PSMA-Ligand PET Imaging

PET/CT and PET/MRI scans were acquired using the Siemens Biograph mCT and the Siemens Biograph mMR (Siemens Healthineers, Erlangen, Germany) in accordance with the EANM/SNMMI guideline for PSMA-ligand PET imaging.

^18^F-rhPSMA7.3 was used in 13 and 7 patients before and after AcPSMA (mean: 305 ± 47 MBq) and LuPSMA (mean: 310 ± 49 MBq), respectively. ^68^Ga-PSMA-11 was used in 8 and 8 patients before and after AcPSMA (mean: 121 ± 22 MBq) and LuPSMA (mean: 106 ± 20 MBq), respectively. Only patients with the pairs of imaging sets with the same radiotracer (^18^F-rhPSMA7.3 or ^68^Ga-PSMA-11) and imaging modality (PET/CT or PET/MRI) were included. 

### 4.3. Image Analysis

The following parameters of the SG were analyzed in all patients to determine the morphological and molecular correlates of its function: a. the morphological volume determined with cross-sectional imaging datasets (Volume-SG), b. the total PSMA-ligand uptake of the SG (PSMA-SGU), which is similar to the total lesion glycolysis determined with ^18^F-FDG PET and represents the total PSMA activity from all tumor voxels [47], and c. SUV_mean_ and SUV_max_ of the SG. d. in patients who underwent ^225^Ac-PSMA-617 RLT, the PSMA-avid tumor volume (PSMA-TV), which is similar to the metabolic tumor volume from ^18^F-FDG PET, was obtained as previously proposed in [47]. All segmentations were performed by one nuclear medicine physician. For all PET-measurements, values were not corrected for body surface or lean body mass.

**Figure 5:**
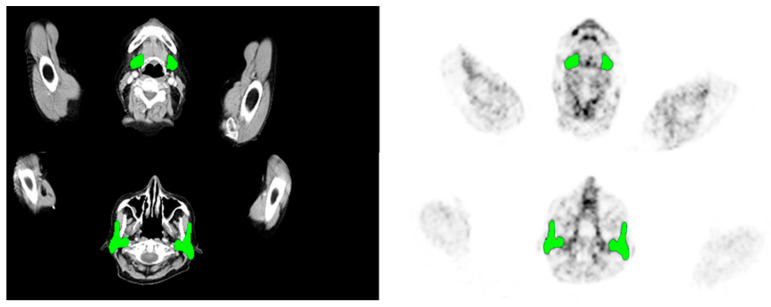
CT based segmentation (left) of submandibular (upper row) and parotid glands (lower row), and the illustration of its transfer to the respective PET images (right). Green colored areas indicate the respective salivary glands.

- Volume-SG was determined in the simultaneously acquired anatomical data (CT or MRI) of the SG. Delineation of the submandibular and parotid glands was measured of each gland separately and on the basis of all available slices (Figure 5).
- PSMA-SGU was quantified before the first and after the first two cycles of LuPSMA (cohort A) treatment and before and after the first cycle of AcPSMA (cohort B). SG was defined as the parotid and the submandibular glands. PSMA-SGU was determined using the in-house developed software qPSMA (with a threshold SUV of 4).
- SUV_mean_ and SUV_max_ was determined using Syngo.Via (Siemens Healthineers, Erlangen, Germany). For SUV_mean_, a 3D VOI using an isocontour of 20% of the SUV_max_ was used.
- PSMA-TV was measured using qPSMA [47]. Bone lesions and soft tissue lesions were separately segmented, and obtained results were summed up. The PSMA-ligand uptake in normal organs was neglected before the quantification of whole-body tumor burden.

### 4.4. Statistical Analysis

To assess the alterations in morphological and functional parameters of the SG after AcPSMA and LuPSMA RLTs, means, standard deviations, and 95% confidence intervals (95%CI) of Volume-SG, PSMA-SG, and SUV_mean_ and SUV_max_ of the salivary glands, and their relative and absolute changes were calculated for cohorts A and B.

To determine the impact of a PSMA positive tumor volume on SG changes in cohort B, PSMA-TV was classified into five groups based on quintiles: very low (Q1: ≤20th percentile), low (Q2: 20th–40th percentile), moderate (Q3: 40th–60th percentile), high (Q4: 60th–80th percentile), and very high (Q5: ≥80th percentile). These quintiles were compared with functional changes in the salivary glands.

T-tests using a two-sided unpaired T-Test with Welch correction were used to compare means of Volume-SG, PSMA-SG, and SUV_mean_ and SUV_max_ of the SG in cohorts A and B and PSMA-TV in cohort B. A *p*-value of <0.05 was considered statistically significant. All calculations were performed using GraphPad Prism version 5.00 (GraphPad Software, San Diego, CA, USA).

## 5. Conclusions

Salivary gland volume and tracer uptake as measured from routine PSMA PET studies are potential biomarker for SG toxicity and should be further evaluated in clinical trials of PSMA radioligand therapy.

## 6. Limitations

One limitation of this retrospective analysis is that it includes both patients with ^68^Ga-PSMA11 and ^18^F-rhPSMA7.3, and this could potentially have an effect on the uptake characteristics of salivary glands. However, we only investigated patients who underwent the same radiotracer pre- and post-treatments, and an additional analysis of our data did not show statistically significant differences in the SUV_max_ and SUV_mean_ in a sub-group analysis both before and after Lu- and Ac-PSMA-RLTs (refer to Supplementary Table S1 and Supplementary Figure S1). Notably, limiting the investigation to only one radiotracer would have substantially reduced the number of suitable patients. Moreover, the direct measurements of the salivary gland function, e.g., using salivary scintigraphy, were not available for analysis in this retrospective analysis.

## Figures and Tables

**Table 1:** Uptake characteristics of salivary glands (SUV_mean_ and SUV_max_) before and after Ac- and Lu-PSMA RLTs of patients from cohort B. Patients are stratified in five groups based on their whole body tumor volume prior to 225Ac-PSMA RLT. Statistically significant changes are marked in bold (* *p* = 0.03, ** *p* = 0.02, *** *p* = 0.04, ^#^ *p* = 0.01).

Whole Body Tumor Volume Prior to AcPSMA		Very Low		Low		Moderate		High		Very High	
		pre	post	pre	post	pre	post	pre	post	pre	post
AcPSMA RLT											
Whole body	PSMA-TV (mL)	602 ± 354	431 ± 296	1393 ± 217	1456 ± 391	1848 ± 156	2370 ± 1076	3378 ± 288	3216 ± 693	4869 ± 342	4296 ± 1252
Salivary glands	SUV_mean_	**11.7 ± 2.4**	6.7 ± 2.4 *	8.1 ± 3.3	4.9 ± 1.1	**11.1 ± 1.8**	5.7 ± 1.6 **	**9.1 ± 2.9**	4.5 ± 2.3 ***	7.8 ± 2.7	5.3 ± 1.7
	SUV_max_	**24.8 ± 4.9**	14.7 ± 4.8 *	17.7 ± 6.1	11.5 ± 2.3	**22.7 ± 3.4**	13.5 ± 2.9 **	**20.9 ± 5.4**	**8.6 ± 1.9 ^#^**	17.3 ± 5.3	12.7 ± 3.2
LuPSMA RLT											
Salivary glands	SUV_mean_	14.9 ± 2.9	13.5 ± 2.6	11.3 ± 2.2	11.2 + 5.3	**9.0 ± 2.8**	11.9 ± 1.9 *	10.2 ± 2.6	8.8 ± 1.4	11.3 ± 2.8	10.3 ± 3.1
	SUV_max_	33.6 ± 9.8	33.4 ± 14.3	23.9 ± 4.0	23.7 ± 10.3	**18.6 ± 4.1**	25.4 ± 5.9 **	21.5 ± 5.3	18.8 ± 2.9	24.5 ± 5.6	26.1 ± 12.1

**Table 2:** Patient characteristics at the timepoint of initiation of ^225^Ac-PSMA RLT.

No.	Number (Agents) of Previous mCRPC Lines Prior to ^225^Ac-RLT	Number (Agents) of Previous mCRPC Lines Prior to ^225^Ac-RLT	Number of LuPSMA Cycles	Activity LuPSMA RLT (GBq)/Cycle	ECOG Score	Metastases	Activity of First AcPSMA RLT (MBq)
1 *	4 (E, A, D, Lu)	4	2	8/7.2	0	B, LN	8
2	8 (D, C, A, C, E, C, Ra, Lu)	8	2	5.7/5.7	0	B	8
3	4 (D, E, A, Lu)	4	8	7.4/7.4/7.3/7.3/7.1/7.1/7.1/7.3	1	B, LN	8
4 *	5 (A, E, Lu, D, Cis/Eto)	5	4	7.2/7.7/7.2/7.7	1	B, LN	8
5 *	6 (D, A, Lu, C, E, Cis/Eto)	6	2	7.6/7.4	1	B, LN, Liver, Lungs	8
6 *	6 (D, Ra, E, C, A, Lu)	6	4	6.9/7.3/7.4/7.5	2	B, LN	10
7	4 (D, Ra, A, Lu)	4	5	7.5/7.3/7.5/7.8/7.7	1	B, LN	8
8 *	8 (CureVac, A + CureVac, D, Study, C, Lu, E, A)	8	2	7.3/7.5	1	B, LN, Lungs	8
9 *	4 (A, D, Lu, E)	4	6	7.2/7.4/7.3/7.4/7.3/6.7	1	B, LN, Peritoneal	10
10 *	3 (A, E, Lu)	3	6	7.3/7.6/7.7/7.0/7.5/ 7.4	1	B, LN	10
11	7 (A, E, D, A, D, C, Lu)	7	6	8.3/7.9/8.3/7.9/7.4/7.3	1	B	8
12	6 (A, E, D, C, Lu, Cis/Eto)	6	2	8.3/7.8	1	B, LN	13
13 *	5 (E, D, A, E, Lu)	5	6	5.1/7.4/7.3/7.6/7.4/6.7	1	B, LN, Liver, Lung	11
14 *	5 (A, E, D, C, Lu)	5	1	7.9	1	B, LN, Liver, Brain	6
15 *	8 (CureVac, A, Ra, Lu, E, D, O, C)	8	6	7.3/7.3/7.3/7.6/7.4/ 7.0	1	B, LN	10
16 *	8 (D, C, A, D, E, A, C, Lu)	8	8	7.3/7.8/7.2/7.2/7.5/7.5/7.5/7.4	0	B, LN, Lungs	12
17 *	5 (D/C, A, D/C, Carbo, Lu)	5	4	7.3/7.6/7.2/7.3	1	LN, B, Peritoneal	9
18	3 (A, Lu, D)	3	5	3.7/3.7/5.5/5.5/4.2	1	B, LN	10
19 *	6 (D, A, E, C, Lu, C)	6	4	6.8/7.6/7.3/9.0	1	B, LN, Liver	14
20 *	5 (E, D, A, Lu, C)	5	4	8.2/7.5/6.2/7.5	1	B, LN	8
21 *	6 (A, E, D, Lu, Ra, C)	6	4	3.3/3.3/3.4/3.5	1	B	8

Abbreviations: Gs = Gleason Score, AP = alkaline phosphatase, LDH = lactate dehydrogenase, AcPSMA = 225Ac-PSMA-617, LuPSMA = 177Lu-PSMA, RLT = radioligand therapy, E = Enzalutamide, A = Abiraterone, D = Docetaxel, Lu = 177Lu-PSMA I&T, RTx = Radiatio, C = Cabazitaxel, Ra = Ra-223-Dichloride, Cis/Eto = Cisplatin/Etoposide, Carbo = Carboplatin; I = immune therapy, O = Olaparib, CureVac = CureVac Study, B = bones, and LN = lymph nodes. * cohort A.

## Data Availability

Data are contained within the article.

## Supplementary Materials

The following supporting information can be downloaded at: https://www.mdpi.com/article/10.3390/ijms242316845/s1.
