# Supplementary material for: Comparative Analysis of Morphological and Functional Effects of 225Ac- and 177Lu-PSMA Radioligand Therapies (RLTs) on Salivary Glands
Source: Int J Mol Sci. 2023 Nov 28;24(23):16845. doi: 10.3390/ijms242316845 (PMC10706561; doi:10.3390/ijms242316845)
Supplement: Supplementary file 1 [file ijms-24-16845-s001.zip › ijms-2615060-supplementary.pdf]

Supplementary Materials

Comparison of PET scans with different PSMA tracers

In order to investigate changes due to different tracers, patients scanned with ^{68}Ga-PSMA11 were compared to patients scanned with ^{18}F-rhPSMA7.3. No statistically significant changes of uptake of SUV_{max} and SUV_{mean} of the salivary glands were observed in scans pre and post Ac and Lu-PSMA-RLT (Supplementary Table S1).

Supplementary Table S1. Analysis of uptake of the salivary glands in patients scanned with either ^{68}Ga-PSMA or ^{18}F-PSMA. No statistically significant changes were observed comparing patients scanned with ^{68}Ga-PSMA to patients scanned with ^{18}F-PSMA pre and post treatment with ^{177}Lu-PSMA and ^{225}Ac-PSMA-617.

SUV_{max}	Lu		Ac	
	pre	post	pre	post
^{68}Ga-PSMA11	22.7±10.7 (n=8)	26.2±12.9 (n=8)	20.3±7.3 (n=8)	13.0±5.0 (n=8)
^{18}F-rhPSMA7.3	25.0±5.2 (n=7)	23.1±9.1 (n=7)	20.0±5.7 (n=13)	11.9±3.4 (n=13)
SUV_{mean}	Lu		Ac	
	pe	post	pre	post
^{68}Ga-PSMA11	10.5±4.2 (n=8)	11.1±3.4 (n=8)	9.4±3.7 (n=8)	5.7±2.4 (n=8)
^{18}F-rhPSMA7.3	11.7±2.8 (n=7)	10.4±4.0 (n=7)	9.1±2.9 (n=13)	5.0±1.7 (n=13)

PSA progression free survival and xerostomia

In a subgroup analysis, we investigated, if patients' xerostomia correlated with PSA progression free survival (PSA PFS) of patients treated with AcPSMA. Patients with no signs of xerostomia, mild impairment (grade 1) and moderate xerostomia (grade 2) were compared with patients that reported high grade (grade 3) xerostomia. PSA progression free survival did not differ between groups with a median PSA PFS of 2.6 ± 2.5 months in grade 1 and 2 xerostomia patients vs. 3.0 ± 2.6 months in patients with grade 3 (Supplementary Figure S1).

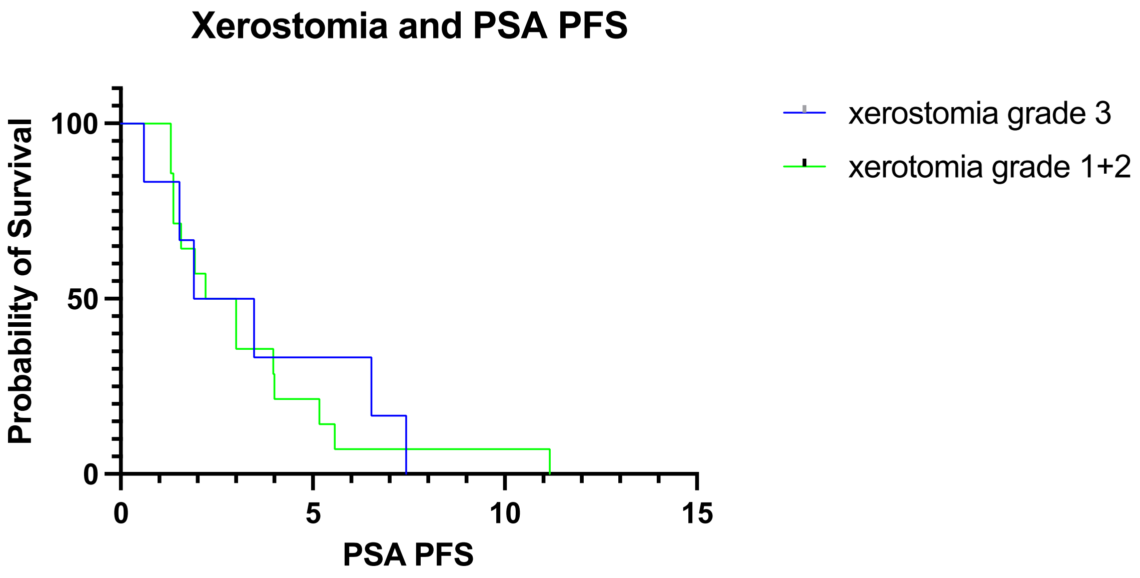

Supplementary Figure S1. Survival Analysis of xerostomia of grade 1 and grade 2 vs. grade 3 in patients treated with AcPSMA showing no difference of PSA progressive free survival in both subgroups.
